# Complete Genome Sequence of *Salmonella enterica* Serovar Typhimurium U288

**DOI:** 10.1128/genomeA.00467-13

**Published:** 2013-07-25

**Authors:** Steven P. T. Hooton, Andrew R. Timms, Joanna Moreton, Ray Wilson, Ian F. Connerton

**Affiliations:** University of Nottingham, School of Biosciences, Division of Food Science, Sutton Bonington Campus, Sutton Bonington, Loughborough, United Kingdoma; DeepSeq, University of Nottingham, Queen’s Medical Centre, Nottingham, United Kingdomb

## Abstract

*Salmonella enterica* serovar Typhimurium U288 has firmly established itself within the United Kingdom pig production industry. The prevalence of this highly pathogenic multidrug-resistant serovar at such a critical point in the food chain is therefore of great concern. To enhance our understanding of this microorganism, whole-genome and plasmid sequencing was performed.

## GENOME ANNOUNCEMENT

*Salmonella enterica* serovar Typhimurium U288 is considered to be a significant pathogen of pigs in the United Kingdom (1). This serovar, which is seemingly adapted to colonization of the pig intestine, is consistently ranked as the number one isolate identified in United Kingdom pig production units over the last decade (2). The prevalence of *S*. Typhimurium U288 in United Kingdom pig herds warrants careful monitoring, as many reported isolates harbor multiple antibiotic resistance determinants, resulting in limited treatment options and potential loss of livestock (3). Furthermore, the consumption of *S*. Typhimurium U288-contaminated pork has been linked to the deaths of several elderly patients in Denmark (4). While *S*. Typhimurium U288 has been the focus of bacteriophage intervention studies (1, 5), there remains a distinct gap in knowledge with regards to the genetic makeup of this pathogen.

For whole-genome sequencing, *S*. Typhimurium U288 genomic DNA was isolated from an overnight NZCYM broth (10 g NZ amine, 5 g Bacto-yeast extract, 5 g NaCl, 1 g Casamino Acids, 1 g MgSO_4_·7H_2_O, dissolved in 1 liter of H_2_O) culture (Difco), incubated at 37°C with shaking, using a GenElute bacterial genomic DNA kit (Sigma-Aldrich, United Kingdom) according to manufacturer’s instructions. Genomic DNA (~5 µg) was fragmented to 500 bp using a Covaris S2 ultrasonicator (Covaris Inc.), and libraries were constructed with NEBNext DNA library master prep mix set 2 (New England Biolabs). Using the Roche 454 GS FLX sequencing system (Roche Diagnostics), a total of 652,721 aligned reads were generated. The aligned reads were *de novo* assembled using CLC Genomics Workbench software (CLC bio, Denmark), generating a total sequence length of 5,017,059 bp. *De novo* contigs were assembled manually and the sequence reads reiteratively mapped to genome drafts using the NextGen tools available in CLC Genomics Workbench. A single contig of 4,852,606 bp (G+C content, 52.18%) representing the complete *S*. Typhimurium U288 chromosome was generated. Sequence reads that did not map to the chromosome were then independently assembled into three circular permuted plasmid DNAs that were ready for annotation. For the primary annotation of assembled chromosomal and plasmid DNA, the NCBI PGAAP (http://www.ncbi.nlm.nih.gov/genomes/static/Pipeline.html) was used, along with a combination of BASys (6) and xBase2 (7). Manual curation of genes and coding sequences (CDSs) in Artemis (8) was performed, with alterations to the finished sequence being made accordingly. The *S*. Typhimurium U288 genome was also scanned for prophage genes using Phast (9). A total of 4,581 CDSs have been identified thus far in the *S*. Typhimurium U288 genome, along with 85 tRNA genes, several rRNA repeats, and 13 putative pseudogenes. A number of prophages dispersed throughout the chromosome were identified as Gifsy 1, Gifsy 2, Fels 2, ST104, and a prophage remnant resembling *Burkholderia* BcepMu. The three plasmids identified in *S*. Typhimurium U288 include a 148,711-bp virulence plasmid (pSTU288-1) containing a class I integron and associated antibiotic resistance cassettes. Further antibiotic resistance determinants are carried on an 11,067-bp plasmid (pSTU288-2). A small 4,675-bp plasmid containing mobilization genes and a gene encoding a GGEEF-domain protein were also identified (pSTU288-3). 

### Nucleotide sequence accession numbers.

The *S*. Typhimurium U288 genome has been deposited in the NCBI database (accession no. CP003836) accompanied by plasmids pSTU288-1, pSTU228-2, and pSTU288-3 (accession no. CP004058, CP004059, CP004060).
